# Study on Laser Transmission Welding Technology of TC4 Titanium Alloy and High-Borosilicate Glass

**DOI:** 10.3390/ma17174371

**Published:** 2024-09-04

**Authors:** Changjun Chen, Lei Li, Min Zhang, Mengxuan Xu, Wei Zhang

**Affiliations:** 1 Laser Processing Research Center, School of Mechanical and Electric Engineering, Soochow University, Suzhou 215131, China; chenchangjun@suda.edu.cn (C.C.); liasnex@outlook.com (L.L.); xmx08201010@163.com (M.X.); 2 State Key Laboratory of Advanced Welding and Joining, Harbin Institute of Technology, Harbin 150001, China; 3 Shi-Changxu Innovation Center for Advanced Materials, Institute of Metal Research, Chinese Academy of Sciences, 62 Wencui Road, Shenyang 110016, China; weizhang@imr.ac.cn

**Keywords:** laser welding, TC4 titanium alloy, glass, microstructure, bonding mechanism

## Abstract

As the demand for high-performance dissimilar material joining continues to increase in fields such as aerospace, biomedical engineering, and electronics, the welding technology of dissimilar materials has become a focus of research. However, due to the differences in material properties, particularly in the welding between metals and non-metals, numerous challenges arise. The formation and quality of the weld seam are strongly influenced by laser process parameters. In this study, successful welding of high-borosilicate glass to a TC4 titanium alloy, which was treated with high-temperature oxidation, was achieved using a millisecond pulsed laser. A series of process parameter comparison experiments were designed, and the laser welding behavior of the titanium alloy and glass under different process parameters was investigated using scanning electron microscopy (SEM) and a universal testing machine as the primary analysis and testing equipment. The results revealed that changes in process parameters significantly affect the energy input and accumulation during the welding process. The maximum joint strength of 60.67 N was obtained at a laser power of 180 W, a welding speed of 3 mm/s, a defocus distance of 0 mm, and a frequency of 10 Hz. Under the action of the laser, the two materials mixed and penetrated into the molten pool, thus achieving a connection. A phase, Ti_5_Si_3_, was detected at the fracture site, indicating that both mechanical bonding and chemical bonding reactions occurred between the high-borosilicate glass and the TC4 titanium alloy during the laser welding process.

## 1. Introduction

Glass, with its excellent oxidation resistance, corrosion resistance, high transparency, and high hardness, is an essential material in the construction, medical, and electronics industries. Metallic materials are widely used in industrial fields because of their strong plasticity, high mechanical strength, and good processability. However, in many cases, the application of a single material no longer meets the requirements of actual production. Therefore, glass needs to be combined with metals that have high toughness and impact resistance. The TC4 titanium alloy, due to its excellent overall performance and good biocompatibility, has been widely used in aerospace, automotive, and biomedical fields [1,2,3]. The combined use of glass and titanium alloys can provide more possibilities for manufacturing and has applications in solar vacuum collectors, smartphone glass covers, and optical devices [4,5,6,7].

There are numerous existing methods for joining glass to metal, such as adhesive bonding [8,9,10,11], mechanical connections [12], brazing [13,14], fusion sealing [15,16], and anodic bonding [17,18,19,20]. However, due to inherent technical flaws or limitations in these methods, traditional joining techniques can no longer meet the demands of the modern, rapidly developing industrial system for high efficiency, high quality, and low pollution. Therefore, it is crucial to find a new welding technology. With the continuous advancement of laser processing technology, its potential for joining glass to metal has garnered extensive attention from researchers.

However, the significant differences in the physical and chemical properties of glass and metal present substantial challenges for their successful welding. Consequently, many researchers have employed advanced ultrafast laser technology for the direct welding of glass and metal [21,22,23,24,25,26], finding that suitable welding process parameters [23,24,26] and focal depth [22,25] are fundamental for the successful welding of these materials. Nonetheless, ultrafast laser systems are expensive. To achieve widespread application of glass–metal composites in actual production, it is still necessary to modify the metal surface [27,28,29] or add intermediate layers [30,31,32,33], in combination with long-pulse laser systems, to achieve reliable and cost-effective bonding of glass and metal.

This study utilized a millisecond-pulsed laser to perform laser transmission welding on high-borosilicate glass and a TC4 titanium alloy treated with high-temperature oxidation. It investigated the effects of laser power, welding speed, defocus distance, and frequency on the weld seam cross-section, fracture morphology, and mechanical properties. The welding mechanism of high-borosilicate glass and the TC4 titanium alloy was elucidated. This research can provide an important theoretical basis for the reliable bonding of glass and metal.

## 2. Materials and Methods

### 2.1. Preparation of Materials

This experiment mainly focused on studying the TC4 titanium alloy (BAOTI Group Co., Ltd., Baoji, China) and high-borosilicate glass (Dongguan Shichuang Glass Co., Ltd., Dongguan, China), with their chemical compositions shown in Table 1 and Table 2, respectively. The titanium alloy substrate for cutting had dimensions of 18 mm × 16 mm × 3 mm, while the customized high-borosilicate glass had dimensions of 18 mm × 14 mm × 1 mm. Due to changes in welding speed and pulse frequency, the overlap rate of the laser spot in the experiment varies from 0% to 75%.

**Figure 1:**
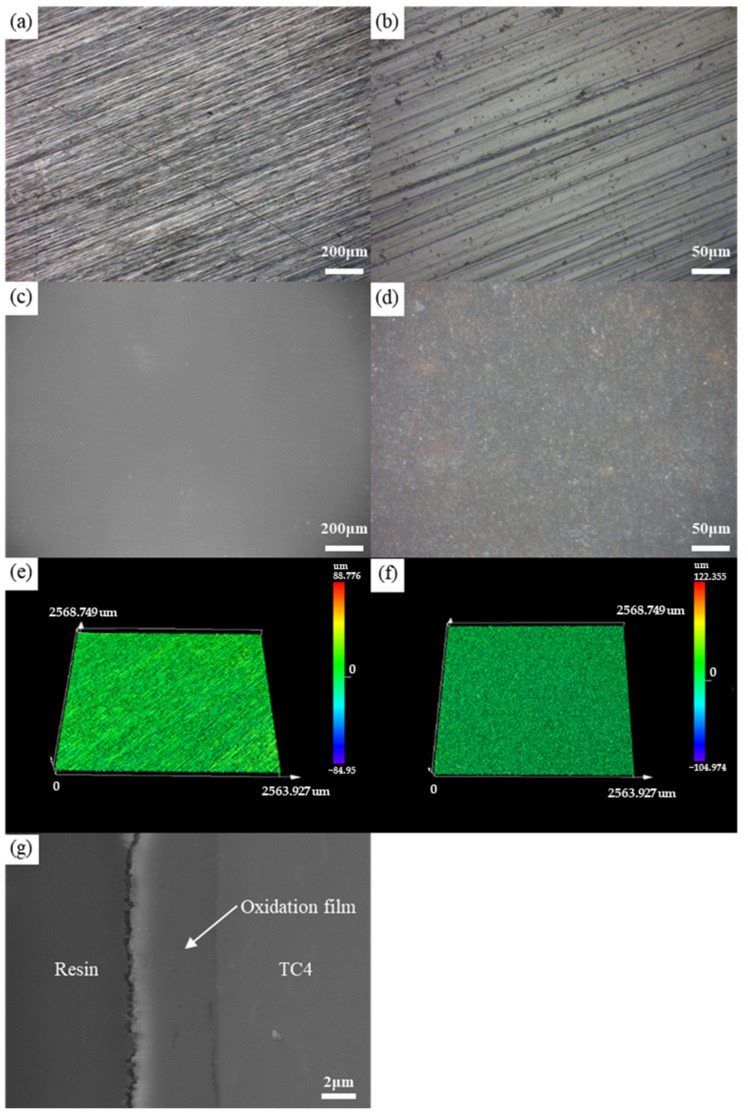
Surface morphology and oxide film cross-section of the TC4 titanium alloy before and after oxidation at 800 °C for 45 min: (**a**,**b**) Surface morphology before oxidation at different magnifications captured by an optical microscope; (**c**,**d**) Surface morphology after oxidation at different magnifications captured by an optical microscope; (**e**) The three-dimensional surface morphology before oxidation captured by a three-dimensional depth-of-field microscope; (**f**) The three-dimensional surface morphology after oxidation captured by a three-dimensional depth-of-field microscope; (**g**) The cross-sectional morphology of the oxide film captured by scanning electron microscopy.

In our previous research [34], it was found that the bonding strength between the TC4 titanium alloy and high-borosilicate glass was highest when the titanium alloy was oxidized at 800 °C for 45 min in a box furnace. The surface morphology of the titanium alloy before and after oxidation, as well as the cross-section of the oxide film, is shown in Figure 1. Therefore, this oxidation parameter was selected for the exploration of laser process parameters in this study.

### 2.2. Welding Experiment

**Figure 2:**
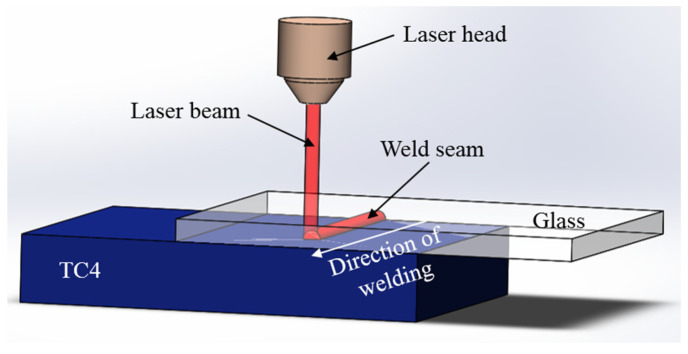
The schematic diagram of laser transmission welding.

The equipment used in this welding experiment is the MD-Focus 300 Nd: YAG laser provided by China Tianhong Laser Co., Ltd. (Suzhou, China). The laser has a wavelength of 1064 nm, a maximum average power of 300 W, a maximum repetition frequency of 100 Hz, pulse widths ranging from 0.1 to 20 ms, and a minimum spot diameter of 0.2 mm. Based on the properties and characteristics of glass and metal, laser transmission welding was adopted for the experiment, as shown in Figure 2. This study systematically investigated the effects of welding speed, power, defocus amount, and frequency on weld quality. To ensure the reliability of the experiments, each part not only examined the effects of a specific parameter but also included additional variations of another parameter for interaction analysis. The aim was to determine the welding parameters that produced the maximum bonding strength. Based on preliminary experiments, suitable parameter ranges were established. Controlled experiments were designed to explore the influence of welding speed, laser power, defocus amount, and frequency on the bonding strength between borosilicate glass and the TC4 titanium alloy. The complete experimental parameter table is shown in Table A1 of Appendix A.

**Figure 3:**
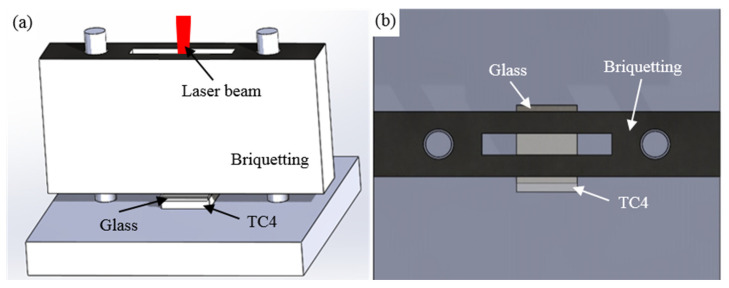
The schematic diagram of the welding fixture: (**a**) Front view; (**b**) Top view.

Some researchers have shown that a significant gap between glass and metal can lead to a decrease in weld quality [35,36,37]. Therefore, to ensure a good bond between glass and metal, it is necessary to design fixtures that provide pre-tensioning to reduce the gap between them. The schematic diagram of the fixture is shown in Figure 3. Since the mass of the pressure block is constant, the additional effect of pre-tensioning force on welding strength is not calculated.

### 2.3. Analysis Method

**Figure 4:**
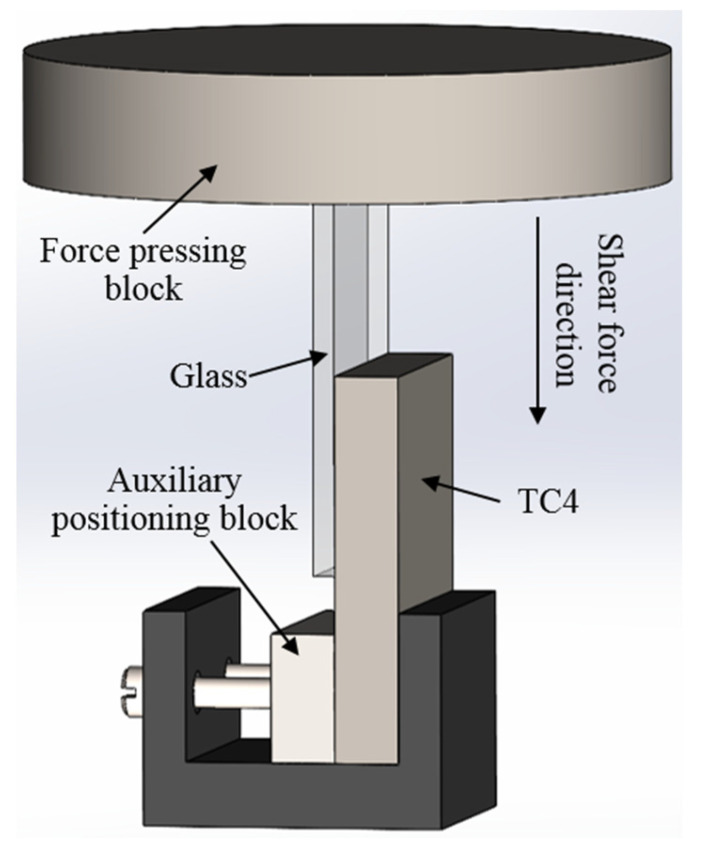
Schematic diagram of the shear force test.

The laser welding was completed and the WDW universal testing machine from Jinan Times Assay Testing Instrument Co., Ltd. in Jinan, China was used to test the bonding strength of the welded parts (five samples were taken for each parameter). A shear fixture, as shown in Figure 4, was designed for the testing process. During the shearing process, the upper pressure block of the system moved downward vertically at a constant speed of 0.2 mm/min. When the glass detached from the metal, indicating the completion of the shearing, the data were recorded on the computer.

The welded joints’ cross-section and fracture morphology were characterized using a scanning electron microscope (SEM, EVO18, Carl Zeiss AG, Oberkochen, Germany). To investigate the bonding mechanism between high-borosilicate glass and the titanium alloy, elemental analysis of the samples was conducted using an energy dispersive spectrometer (EDS). Additionally, phase detection was performed on the fractured surfaces of the welded joints. The scanning range for the tests was set at 10–80°, with a scanning speed of 5°/min.

## 3. Results and Discussion

### 3.1. Impact of Welding Speed on Welded Joints

**Figure 5:**
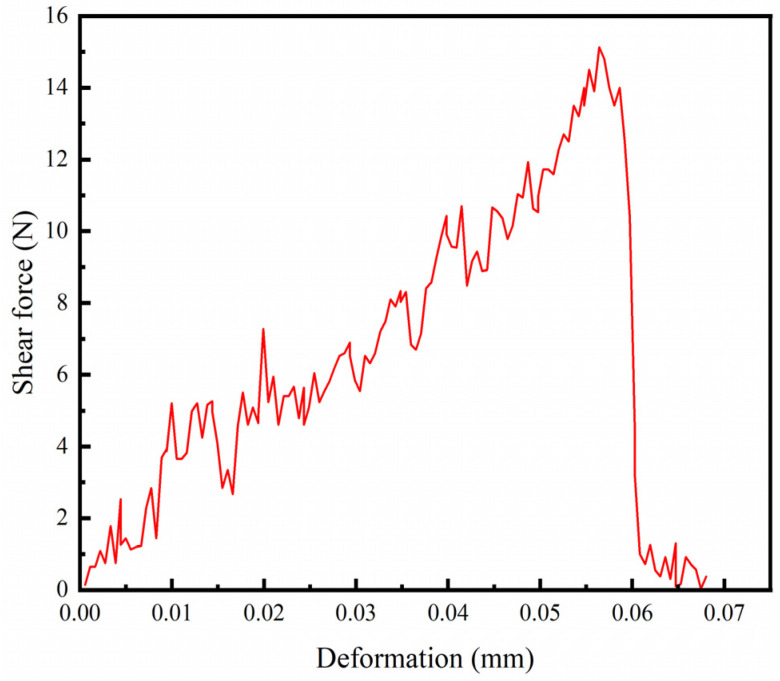
Curve of Shear force deformation (power: 180 W, welding speed: 1 mm/s, defocus distance: 0 mm, pulse frequency: 10 Hz).

**Figure 6:**
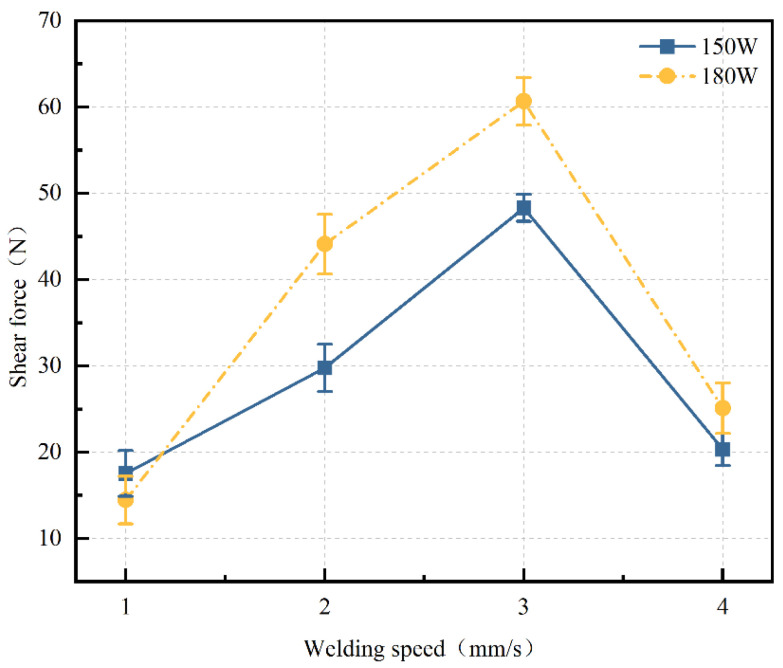
Curve of shear force changing with welding speed (defocus distance: 0 mm, pulse frequency: 10 Hz. They all remained constant).

Welding speed refers to the rate at which the laser beam moves across the surface of the specimen during the welding process. To ensure the reliability of the experimental results, shear strength tests were conducted at different welding speeds (1 mm/s, 2 mm/s, 3 mm/s, and 4 mm/s) for varying power levels (150 W and 180 W), while other process parameters, including defocus distance (0 mm) and frequency (10 Hz), were kept constant. Figure 5 shows the shear force–deformation curve at a power of 180 W and a welding speed of 1 mm/s, with a maximum shear force of 15.13 N. The variation of shear force with welding speed for different power levels is presented in Figure 6. At power levels of both 150 W and 180 W, the shear force of the welded joints initially increases and then decreases as the welding speed increases. The maximum shear force, approximately 60.67 N, is observed at a laser power of 180 W and a welding speed of 3 mm/s. At a welding speed of 1 mm/s, the shear force values for both power levels were very low, likely due to the accumulation of heat at such a low welding speed, leading to welding defects. However, excessively high welding speeds can reduce the time the laser beam acts on the sample surface, resulting in reduced energy and potentially incomplete welding or poor joint strength. A welding speed of 3 mm/s provides a relatively high welding speed, which can reduce the heat-affected zone and thermal stress while controlling appropriate heat input. Therefore, at power levels of 150 W and 180 W, a welding speed of 3 mm/s can achieve good joint strength between the welded joints of high-borosilicate glass and the TC4 titanium alloy.

**Figure 7:**
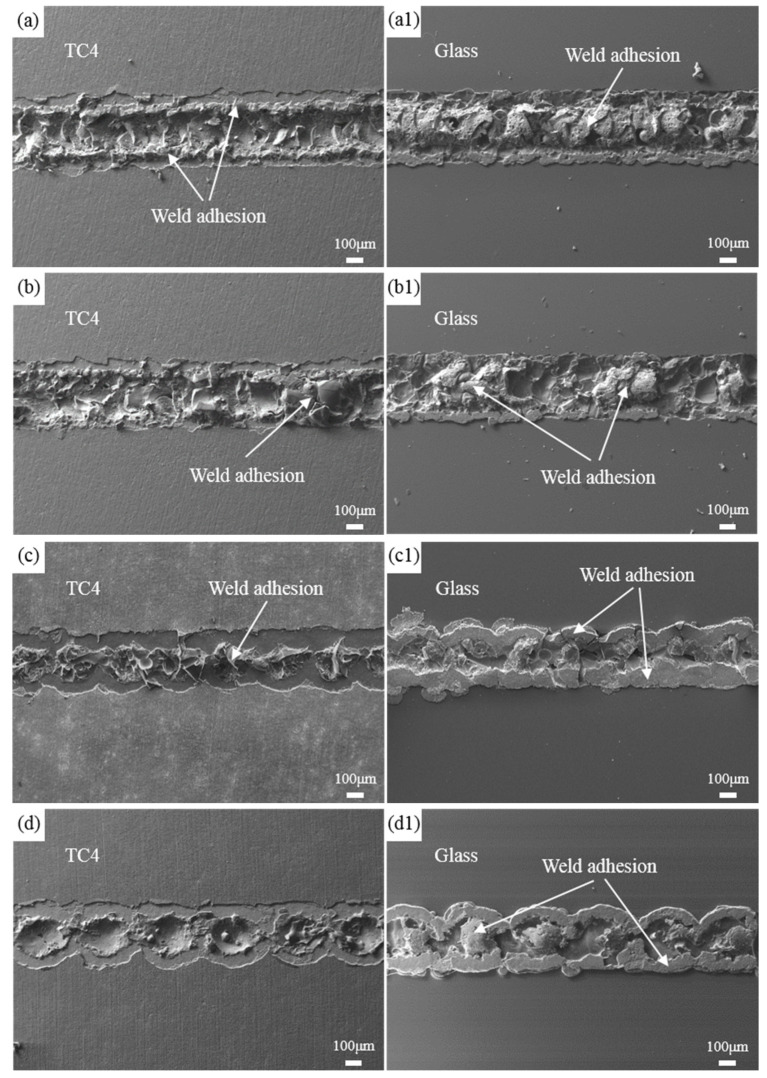
The fracture morphology of welded joints at different welding speeds. (**a**–**d**) Metal side; (**a1**–**d1**) Glass side; (**a**) 1 mm/s; (**b**) 2 mm/s; (**c**) 3 mm/s; (**d**) 4 mm/s.

Figure 7 presents the fracture morphology of the welded joints after shear testing at different welding speeds under a laser power of 180 W. It can be observed from the Figure that the weld seam width gradually decreases with increasing welding speed. This is because, at lower speeds, the laser exposure time is longer, allowing the metal to absorb more energy, resulting in a larger melt volume in the welding area. At a welding speed of 4 mm/s, due to the high speed, each laser pulse can be observed at the fracture location, and there is almost no residue on the metal side after shearing, with only a small amount of adhesive material on the glass side. These small amounts of adhesive material mainly consist of oxide film peeled off from the closed zone at both ends of the weld seam, which matches the fracture morphology of the weld seam on the metal side. This indicates that at high welding speeds, insufficient heat input leads to poor mechanical properties in the bonding zone formed by the melting of glass and metal. At a welding speed of 1 mm/s, the excessively low speed leads to greater heat accumulation, resulting in excessive stress on the metal side, causing the welded joint to fracture from the metal side. At a welding speed of 2 mm/s, fractures occur on both the glass and metal sides of the welded joint. As shown in Figure 6, the shear force values of the welded joints under these conditions are relatively low. Only at a welding speed of 3 mm/s does the welded joint first fracture near the oxide film in the weld seam closure area, followed by a complete fracture within the molten pool. In this case, a reliable bond between the borosilicate glass and titanium alloy can be achieved.

### 3.2. Impact of Power on Welded Joints

**Figure 8:**
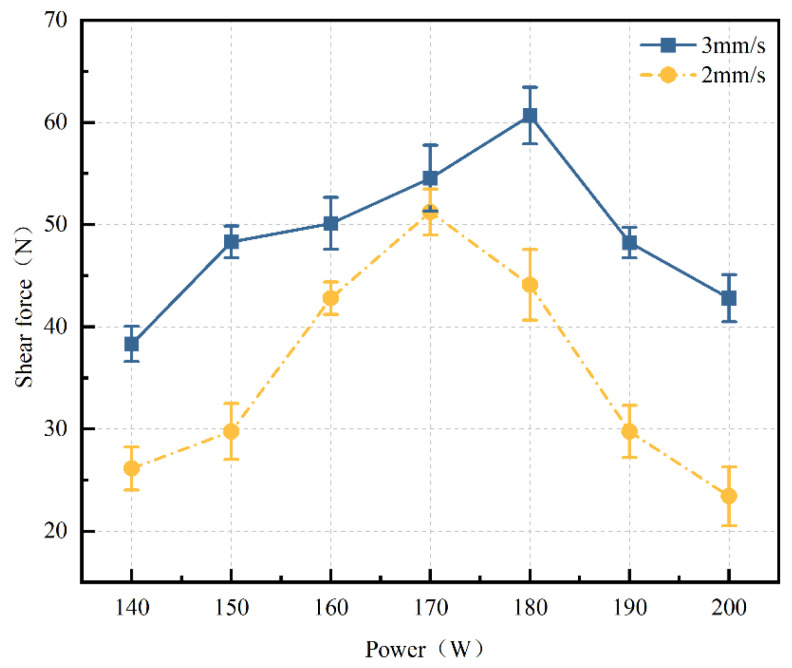
Curve of shear force changing with power (defocus distance: 0 mm, pulse frequency: 10 Hz. They all remained constant).

The laser used in this experiment is a pulsed laser, with laser power referring to the average power in the text. The experiment found that laser power plays a crucial role in the bonding strength of the welded joints. In this study, shear force tests were conducted at different welding speeds (2 mm/s and 3 mm/s) under varying power levels (140 W to 200 W), with other process parameters, such as defocus distance (0 mm) and frequency (10 Hz), kept constant. The resulting shear force versus power curves are shown in Figure 8. From the Figure, it can be seen that at both welding speeds, the shear force initially increases and then decreases as the power increases. The maximum bonding strength of 60.67 N is achieved at a power of 180 W and a welding speed of 3 mm/s. This is because, with other process parameters remaining unchanged, increasing the laser power enhances the energy output per unit of time, leading to an increased melt volume of the glass and TC4 titanium alloy, thereby increasing the shear force within a lower power range. When the laser power is too low, the melting of the material is insufficient, preventing a strong bond from forming. However, excessively high power may cause material overheating and damage, resulting in a decrease in shear force when the power is increased from 180 W to 200 W. Within the power range of 140 W to 200 W, the shear force at a welding speed of 2 mm/s is generally lower than that at 3 mm/s. As previously analyzed, this is because the lower welding speed generates excessive thermal stress, leading to poorer bonding performance of the welded joints.

**Figure 9:**
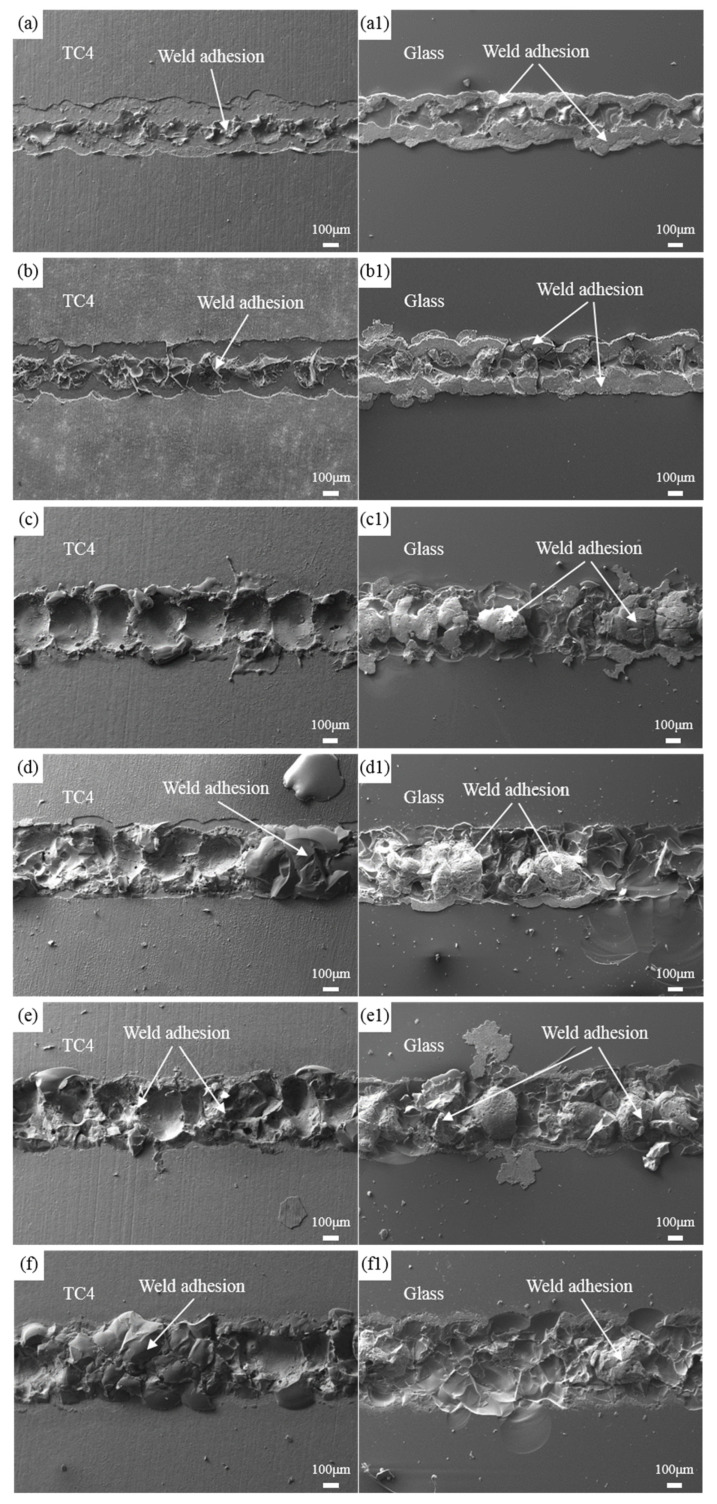
The fracture morphology of welded joints at different power levels. (**a**–**f**) Metal side; (**a1**–**f1**) Glass side; (**a**) 140 W; (**b**) 150 W; (**c**) 160 W; (**d**) 170 W; (**e**) 180 W; (**f**) 190 W.

The fracture morphology of the welded joints after shear testing at different laser power levels with a welding speed of 3 mm/s is shown in Figure 9. It can be observed that as the power increases, the weld seam width continuously increases. At a power of 140 W, the laser energy output is low, resulting in shallow melting depths on both the titanium alloy and glass sides. The residues on both sides are primarily metal surface oxide films, with very little material in the weld pool. With increasing power, more energy is input into the material, expanding the width and depth of melting for both the glass and metal sides, leading to an increase in the amount of residues on both sides. At 160 W, the welded joint fractures from the metal side; at 170 W, fractures occur from both the metal and glass sides, with a small number of fractures observed from within the weld pool, as shown in Figure 9(d,d1). At 180 W, residues can be observed on both the glass and metal sides of the weld seam, without obvious tearing on either glass or metal surfaces, indicating fracture from within the weld pool. However, unlike at 150 W, there is a noticeable reduction in the peel-off area of oxide films on both sides of the weld seam, indicating a reliable bond formed in the closed zone at both ends of the weld seam at 180 W, resulting in the highest shear force value for this parameter setting. At 190 W, most residues are left on the metal side of the weld seam, with clear fracture lines on the glass side, corresponding to a decrease in shear force values.

### 3.3. Impact of Defocus Distance on Welded Joints

**Figure 10:**
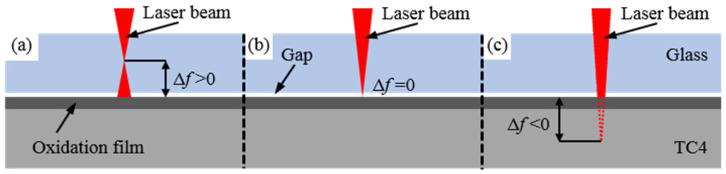
The focal point of the laser beam at different defocus distances. (**a**) Positive defocus (Δf > 0); (**b**) Zero defocus (Δf = 0); (**c**) Negative defocus (Δf < 0).

**Figure 11:**
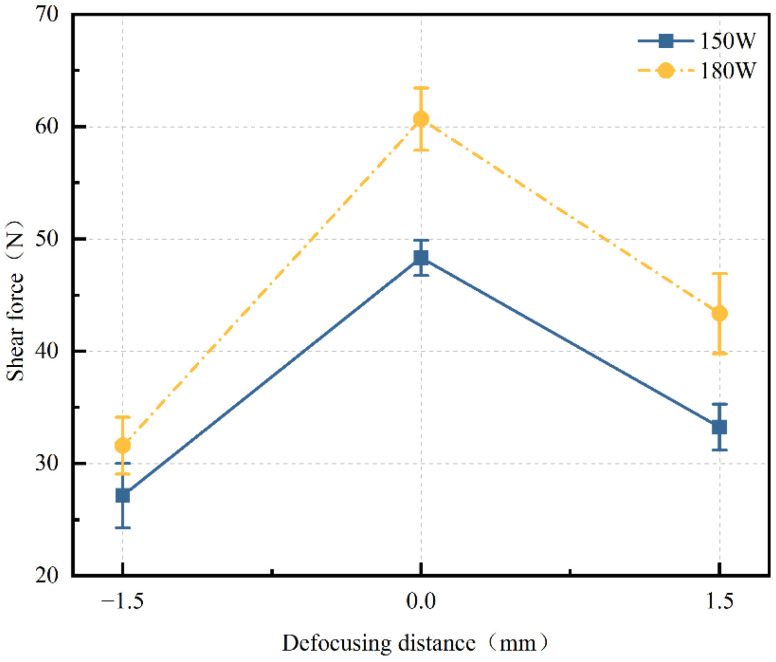
Curve of shear force changing with defocus (welding Speed: 3 mm/s, pulse frequency: 10 Hz. They all remained constant).

Defocus distance is a crucial parameter in laser welding, referring to the distance between the focal point of the laser beam and the joint surface of the welded component, as shown in Figure 10. Shear force tests were conducted at different power levels (150 W and 180 W) with varying defocus distances (−1.5 mm, 0 mm, and +1.5 mm) while keeping other process parameters constant: a welding speed of 3 mm/s and a frequency of 10 Hz. The resulting curves of shear force versus defocus distance for different power levels are presented in Figure 11. From the graph, it can be observed that as the focal point of the laser beam moves from the metal side towards the glass side, the shear force of the welded joints first increases and then decreases. The best welding results for both power levels are achieved at a defocus distance of 0 mm. This is because changing the defocus distance can alter the focusing effect of the laser beam and the distribution of energy density. At a defocus distance of 0 mm, the laser energy distribution is more concentrated, allowing for better melting and bonding of the upper and lower layers of materials. However, when the defocus distance is +1.5 mm or −1.5 mm, a larger focal spot is formed on the welded joint, resulting in a more dispersed energy distribution on the welded component. This reduces the heat input in the welding area, leading to weaker weld seams. Therefore, excessively large or small defocus distances may result in decreased welding quality. Only by selecting an appropriate defocus distance can optimal bonding be achieved.

**Figure 12:**
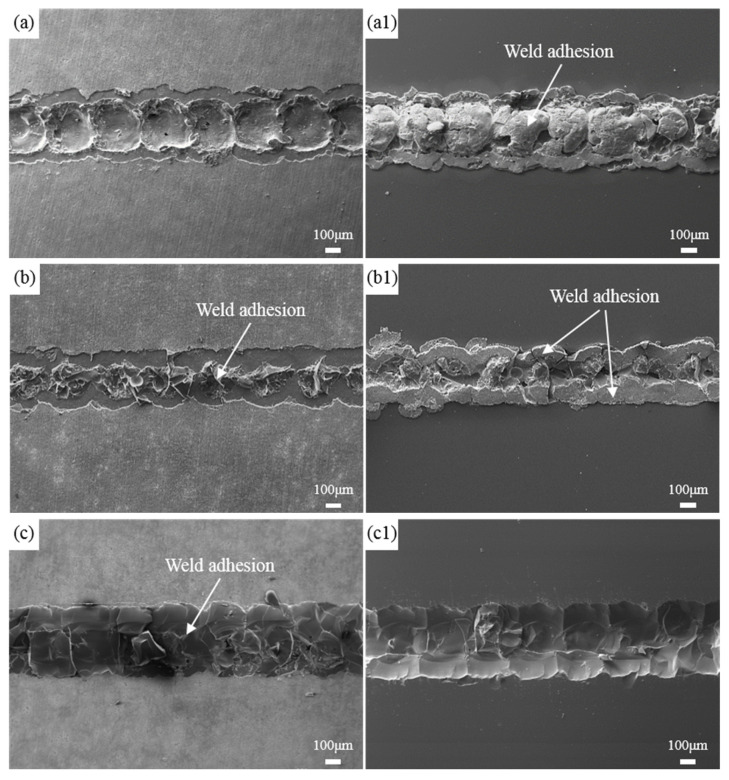
Fracture morphology of welded joints at different defocus distances. (**a**–**c**) Metal side; (**a1**–**c1**) Glass side; (**a**) +1.5 mm; (**b**) 0 mm; (**c**) −1.5 mm.

The continuous variation of the defocus distance results in significant differences in the fracture morphology of the welded joints between high-borosilicate glass and the TC4 titanium alloy, as shown in Figure 12. From the graph, it can be observed that when the focal point of the laser beam is positioned above the joint between the glass and metal, as the focal point shifts downward towards the interface and then further down below the interface (+1.5 mm to −1.5 mm), residues of the weld seam gradually appear at the fracture on the metal side, while the residues on the glass side of the weld seam decrease. When the defocus distance is +1.5 mm or −1.5 mm, the welded joint fractures from the metal side and glass side, respectively, with a clear boundary, indicating a low bond strength under these two parameters.

### 3.4. Impact of Frequency on Welded Joints

**Figure 13:**
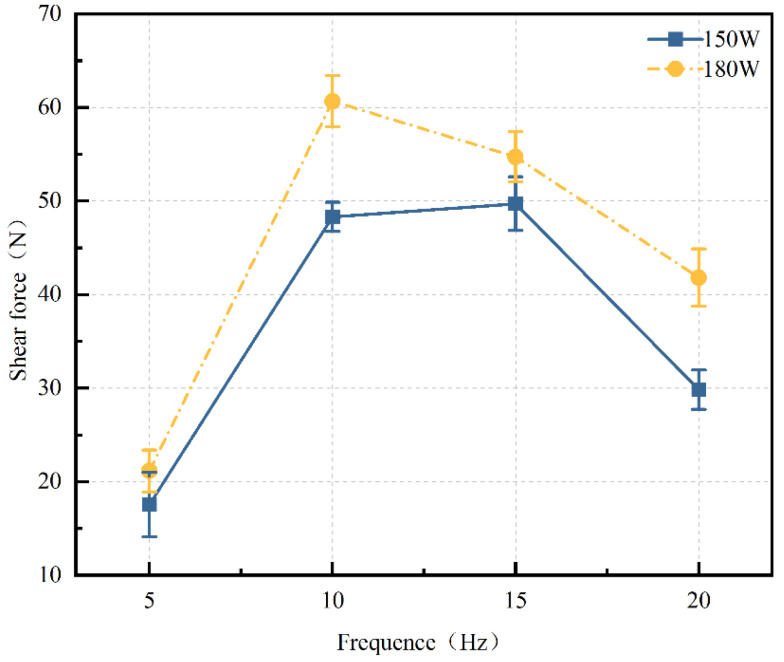
Curve of shear force changing with frequency (welding Speed: 3 mm/s, defocus distance: 0 mm. They all remained constant).

Frequency refers to the number of pulses repeated per unit of time, and its relationship with power is shown in Equation (1) [38]:

(1) $$P=E_{p}f$$

where *P* is the average power (W), *E_p_* is the pulse energy (J) and *f* is the frequency (Hz). Shear force tests were conducted at different power levels (150 W and 180 W) with varying frequencies (5 Hz, 10 Hz, 15 Hz, and 20 Hz), while keeping other process parameters constant: a welding speed of 3 mm/s and a defocus distance of 0 mm. The results are shown in Figure 13. At both 150 W and 180 W, the shear force of the welded joints initially increases and then decreases with the increase in laser frequency. The highest shear force, approximately 60.67 N, is achieved at a laser power of 180 W and a frequency of 10 Hz, indicating that both too low and too high a frequency can result in poor welding performance. As shown in Equation (1), for a constant average laser power P, a lower pulse frequency leads to higher single-pulse energy, resulting in excessive heat input that can damage the welded joints. Conversely, a higher pulse frequency results in lower single-pulse energy, which may be insufficient to melt, diffuse, and bond the glass and metal at the interface. Additionally, at high frequencies, the pulse overlap rate is very high, leading to repeated welding and a rapid decrease in shear force. Therefore, this experiment concludes that the bonding performance of the welded joints is optimal within the frequency range of 10 Hz to 15 Hz.

**Figure 14:**
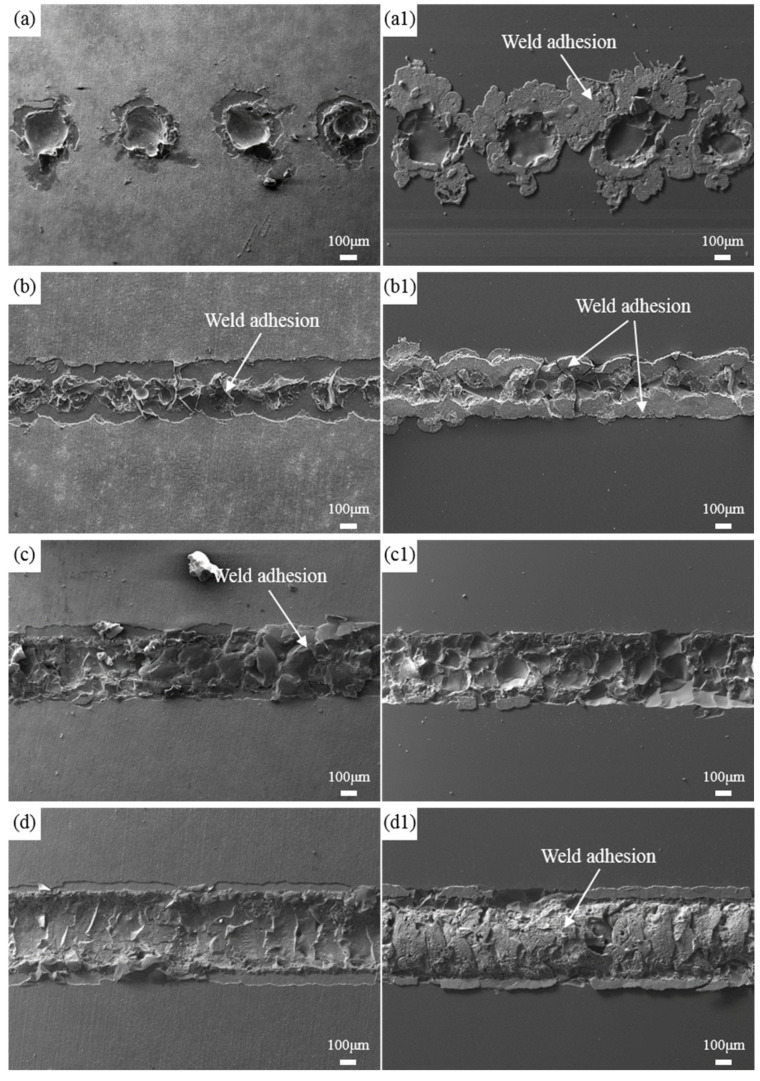
Fracture morphology of welded joints at different frequencies. (**a**–**d**) Metal side; (**a1**–**d1**) Glass side; (**a**) 5 Hz; (**b**) 10 Hz; (**c**) 15 Hz; (**d**) 20 Hz.

The fracture morphology of both the glass and titanium alloy sides at frequencies of 5 Hz, 10 Hz, 15 Hz, and 20 Hz is shown in Figure 14. From Figure 14(a,a1), it can be observed that at 5 Hz, the pulse spacing at the weld seam between the glass and metal sides is large, resulting in a discontinuous weld seam. The metal side shows almost no weld residue, while the glass side mainly exhibits residuals of oxide films and weld spatter originating from the metal side. As previously noted, with constant average laser power, a lower frequency results in higher single-pulse energy, leading to spattering of the molten pool due to the impact of the laser shockwave, as seen in Figure 14(a1). This causes thermal damage to the weld seam, and no adhesion at the weld seam center is observed on either the glass or metal side, resulting in poor bonding strength, consistent with the low shear force values in Figure 13. At a frequency of 10 Hz, a continuous weld seam is formed. At 15 Hz, only a small amount of oxide film is observed on both sides of the glass weld seam, indicating the formation of a reliable closure zone on both sides of the weld seam, which corresponds to a higher shear force value under this parameter. When the frequency increases to 20 Hz, the fracture morphology reveals significant pulse overlap, with a noticeable increase in weld seam width. On the metal side, tear surfaces corresponding to each pulse impact point can be observed. The glass side is covered with adherent material at the weld seam, indicating that the joint fractured from the metal side. The corresponding shear force value is lower at 20 Hz because the reduced single-pulse energy, combined with the high pulse overlap rate, results in multiple welds at the same location, leading to excessive energy accumulation and significant thermal damage to the welded joint.

### 3.5. Welding Mechanism of Titanium Alloy and Glass

**Figure 15:**
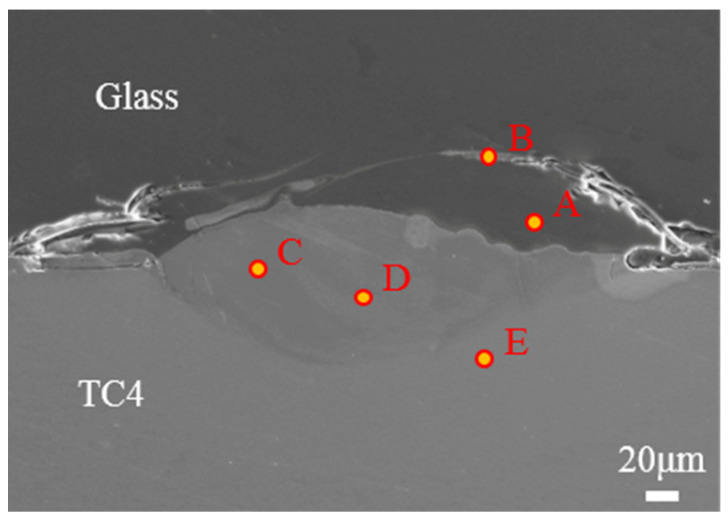
The cross-sectional morphology of the welded joint under the conditions of 150 W power, welding speed of 3 mm/s, defocus of −1.5 mm, and frequency of 10 Hz; A and B are located on the glass side; C and D are located on weld pool; E is located on titanium alloy side.

**Figure 16:**
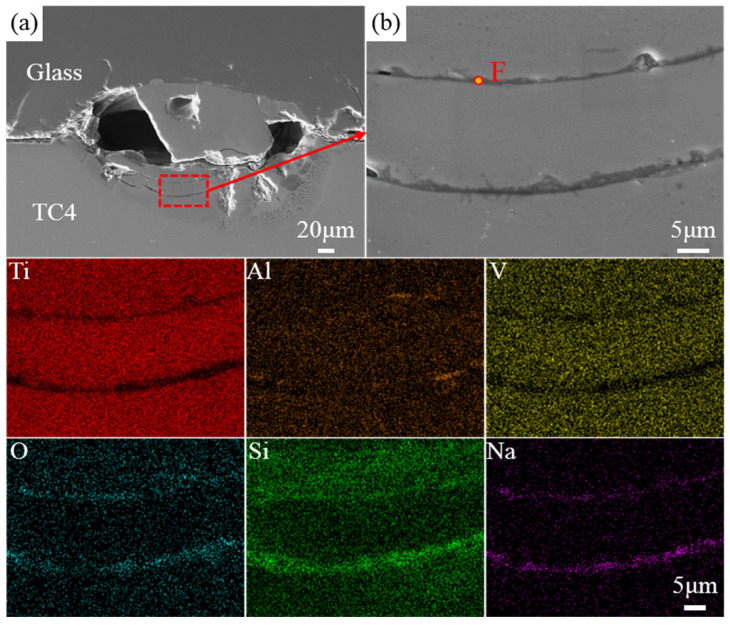
The EDS spectra of the cross-sectional morphology of the welded joint under the conditions of 150 W power, welding speed of 1 mm/s, defocus of 0 mm, and frequency of 10 Hz, including (**a**) the overall view and (**b**) the locally magnified image. F is located on weld pool.

For the elemental composition analysis of the weld pool, two typical weld cross-sections were characterized, as shown in Figure 15 and Figure 16, and EDS analysis was conducted on several typical positions in and around the weld pool. The corresponding spectrum results are shown in Table 3. Region A is located on the glass side, so it contains only four elements: Si, O, Al, and Na. Region E is on the titanium alloy side, so it contains only three elements: Ti, Al, and V. Points C and D are located inside the weld pool, and although the mass percentages of elements differ slightly between them, both points contain elements from both the glass and the metal [29]. This phenomenon is consistent with what previous scholars have observed. Additionally, Figure 15 shows that the boundary in the central welding zone is not distinct, indicating a connection formed through a mixture of glass and metal under the thermal melting and thermal impact of the laser. Some ribbon-like features resembling the weld pool are observed on the glass side above the weld pool, and the spectrum at point B indicates that these areas are also mixtures of glass and metal. This suggests that under laser action, not only uniform penetration and diffusion of glass and metal occur within the weld pool but also intense compression splashing.

In Figure 16a, two semicircular-like crack-like substances are observed on the metal side of the weld pool. Local magnification and elemental analysis were performed on these areas. From the elemental distribution map, it can be seen that Si, O, and Na are enriched at the two curves, indicating that these are not cracks but rather glass components. Local point scans were conducted on the curves, and the spectrum results at point F show that oxygen (O) and silicon (Si) together account for about 90% of the total composition, while titanium (Ti) content is only about 1.7%. This indicates that under laser action, not only do glass and metal melt and diffuse into each other but a small amount of glass is also directly embedded into the metal due to compression without significant penetration, resulting in a distinct interface difference between them.

**Figure 17:**
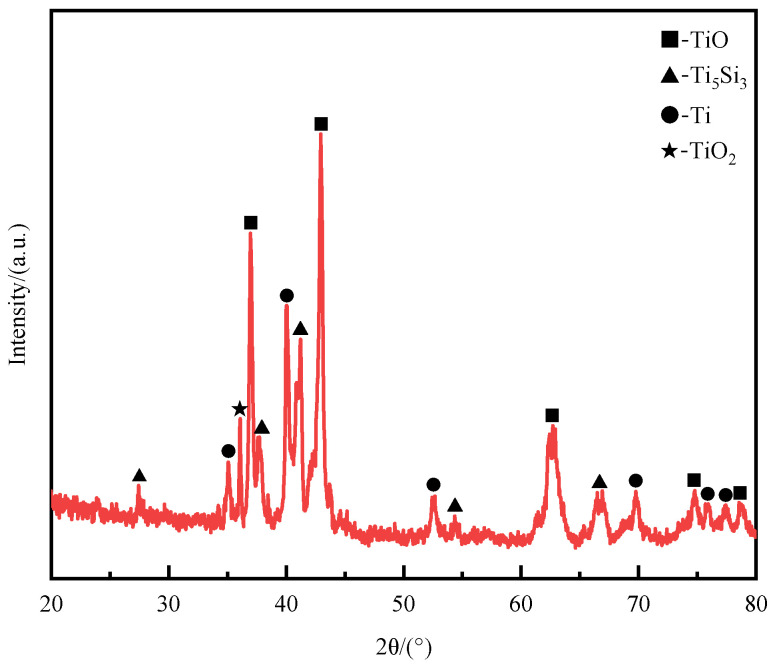
X-ray diffraction pattern of the weld seam.

To further explore the bonding mechanism between high-borosilicate glass and the TC4 titanium alloy, it is necessary to understand the phase changes at the weld. Therefore, X-ray detection was performed on the metal side fracture of the welded component after shear testing, and the results are shown in Figure 17. From the phase results, several phases were detected in the weld, including TiO, Ti_5_Si_3_, Ti, and TiO_2_. Among them, TiO_2_ is the main phase composition of the titanium alloy surface oxide film. Since the weld does not cover the titanium alloy substrate surface completely, a certain amount of Ti was also detected. TiO and Ti_5_Si_3_ are phases that do not exist in either the titanium alloy or the high-borosilicate glass, indicating that under laser action, the titanium alloy underwent thermal oxidation to form the new phase TiO, and a chemical reaction occurred between the high-borosilicate glass and the titanium alloy during the element fusion process, resulting in the new phase Ti_5_Si_3_. Therefore, it is proven that high-borosilicate glass and the TC4 titanium alloy not only mechanically bond during laser welding but also undergo chemical bonding reactions.

**Figure 18:**
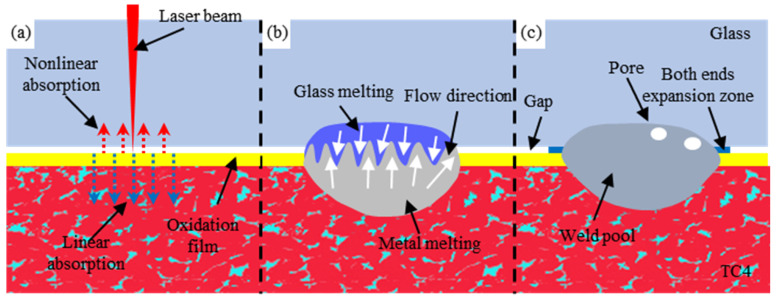
The schematic diagram of the formation process of the weld seam between high-borosilicate glass and the TC4 titanium alloy. (**a**) Laser irradiation phase; (**b**) Material melting and mixing phase; (**c**) Material forming phase.

Figure 18 depicts the schematic diagram of the welding process between high-borosilicate glass and the TC4 titanium alloy. Due to the strong transmittance of glass to laser light, the laser beam focus acts on the metal surface under laser radiation. The majority of the energy is transmitted through the oxide film to be linearly absorbed by the metal inside, while a portion is nonlinearly absorbed by the glass due to refraction. With the continuous action of the laser, the metal absorbs energy, causing the upper and lower layers of material to reach a molten state. This leads to reliable bonding by local modification at high temperatures and rapid cooling and solidification to fill the gap between them. Additionally, during the mixing process in the molten pool, some molten liquid also expands due to thermal pressure and flows into adjacent interface gaps, as shown in the expanded areas at both ends in Figure 18c. Therefore, the interface gaps in the unirradiated areas are also filled and connected.

**Figure 19:**
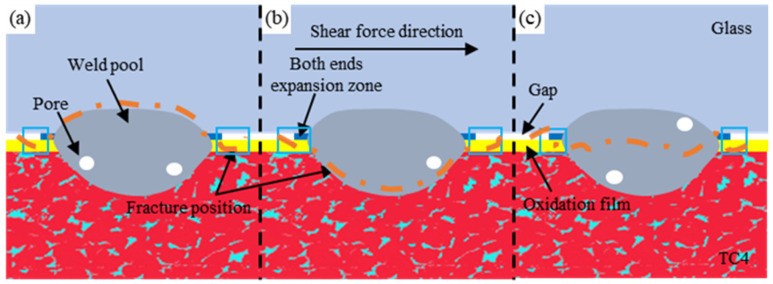
Schematic diagram of the shear fracture of the high-borosilicate glass and the TC4 titanium alloy welding joint. (**a**) Fracture mode one; (**b**) Fracture mode two; (**c**) Fracture mode three.

Under different laser process parameters, the weld seams between high-borosilicate glass and the TC4 titanium alloy fracture from different positions during shear testing, which is mainly divided into three fracture modes as shown in Figure 19. When the laser energy is low, it fails to provide sufficient heat to fully melt the glass and metal, resulting in a smaller bonding area and causing the welding joint to fracture from either the glass substrate or the metal substrate side, as shown in Figure 19a,b. Consequently, the corresponding welding strength is relatively weak. Conversely, excessive laser energy can create defects such as cracks and voids, as shown in Figure 16a, despite forming a larger weld seam between the glass and metal. This leads to unreliable welding results and fractures occurring at either the glass substrate or metal substrate side. When the laser energy is moderate, a good penetration bond is formed between the high-borosilicate glass and the titanium alloy within the weld pool. In this case, the welding joint fractures from the middle of the weld pool under shear stress, as depicted in Figure 19c. At this point, the joint’s resistance to external forces is strongest, resulting in the optimal bonding effect. Additionally, as the laser action concludes, stress concentrations occur during the cooling process, causing the oxide film near the weld pool to shrink and cracks to form in the titanium alloy substrate. This leads to fractures occurring first at the junction between the oxide film and the substrate, as shown in the red box area in Figure 19.

## 4. Conclusions

(1) By adjusting the process parameters, appropriate heat input can be controlled. When the laser power is 180 W, welding speed is 3 mm/s, focal offset is 0 mm, and frequency is 10 Hz, the maximum joint strength of the welded component reaches 60.67 N. Suitable process parameters help improve the bonding strength between the glass and metal.
(2) Glass and metal are diffused and infiltrated in the molten pool or directly embedded in each other under the action of laser thermal melting and thermal shock, which is the reason why the two materials can be combined.
(3) Under the action of the laser, a chemical reaction occurred between high-borosilicate glass and the TC4 titanium alloy, producing a new phase Ti_5_Si_3_. This indicates that during laser welding, not only mechanical bonding but also chemical bonding reactions occur between the two materials.

## Figures and Tables

**Table 1:** Element content in the TC4 titanium alloy (mass fraction/%).

Ti	Fe	C	N	H	O	Al	V
>99	≤0.3	≤0.1	≤0.05	≤0.015	≤0.2	5.5–6.8	3.5–4.5

**Table 2:** Chemical composition of high-borosilicate glass (mass fraction/%).

SiO_2_	B_2_O_3_	Al_2_O_3_	Na_2_O and K_2_O	Other
80.4	12.7	2.4	4.2	0.3

**Table 3:** EDS energy spectrum analysis of each point in Figure 13 and Figure 14 (mass percentage/%).

	Ti	Al	V	O	Si	Na	Other
A	/	1.4	/	50.9	43.6	1.8	2.3
B	41.1	5.7	1.2	38.1	11.9	1.0	1.0
C	32.2	3.4	3.0	40.0	15.3	1.5	4.6
D	42.8	3.7	2.5	34.9	10.2	3.8	2.1
E	88.2	5.8	4.4	/	/	/	1.6
F	1.7	1.5	/	50.0	40.5	3.1	3.2

## Data Availability

The raw data supporting the conclusions of this article will be made available by the authors on request.

## Appendix A

**Table A1:** Experimental Parameter Table.

Experiment Number	Welding Speeds	Power	Defocus Distance	Pulse Frequency
1	1 mm/s	150 W	0 mm	10 Hz
2	1 mm/s	180 W	0 mm	10 Hz
3	2 mm/s	150 W	0 mm	10 Hz
4	2 mm/s	180 W	0 mm	10 Hz
5	3 mm/s	150 W	0 mm	10 Hz
6	3 mm/s	180 W	0 mm	10 Hz
7	4 mm/s	150 W	0 mm	10 Hz
8	4 mm/s	180 W	0 mm	10 Hz
9	1 mm/s	180 W	−1.5 mm	10 Hz
10	1 mm/s	180 W	1.5 mm	10 Hz
11	2 mm/s	180 W	−1.5 mm	10 Hz
12	2 mm/s	180 W	1.5 mm	10 Hz
13	3 mm/s	180 W	−1.5 mm	10 Hz
14	3 mm/s	180 W	1.5 mm	10 Hz
15	4 mm/s	180 W	−1.5 mm	10 Hz
16	4 mm/s	180 W	1.5 mm	10 Hz
17	1 mm/s	180 W	0 mm	5 Hz
18	1 mm/s	180 W	0 mm	15 Hz
19	1 mm/s	180 W	0 mm	20 Hz
20	2 mm/s	180 W	0 mm	5 Hz
21	2 mm/s	180 W	0 mm	15 Hz
22	2 mm/s	180 W	0 mm	20 Hz
23	3 mm/s	180 W	0 mm	5 Hz
24	3 mm/s	180 W	0 mm	15 Hz
25	3 mm/s	180 W	0 mm	20 Hz
26	4 mm/s	180 W	0 mm	5 Hz
27	4 mm/s	180 W	0 mm	15 Hz
28	4 mm/s	180 W	0 mm	20 Hz
29	1 mm/s	140 W	0 mm	10 Hz
30	1 mm/s	160 W	0 mm	10 Hz
31	1 mm/s	170 W	0 mm	10 Hz
32	1 mm/s	190 W	0 mm	10 Hz
33	1 mm/s	200 W	0 mm	10 Hz
34	2 mm/s	140 W	0 mm	10 Hz
35	2 mm/s	160 W	0 mm	10 Hz
36	2 mm/s	170 W	0 mm	10 Hz
37	2 mm/s	190 W	0 mm	10 Hz
38	2 mm/s	200 W	0 mm	10 Hz
39	3 mm/s	140 W	0 mm	10 Hz
40	3 mm/s	160 W	0 mm	10 Hz
41	3 mm/s	170 W	0 mm	10 Hz
42	3 mm/s	190 W	0 mm	10 Hz
43	3 mm/s	200 W	0 mm	10 Hz
44	4 mm/s	140 W	0 mm	10 Hz
45	4 mm/s	160 W	0 mm	10 Hz
46	4 mm/s	170 W	0 mm	10 Hz
47	4 mm/s	190 W	0 mm	10 Hz
48	4 mm/s	200 W	0 mm	10 Hz
49	3 mm/s	140 W	0 mm	5 Hz
50	3 mm/s	140 W	0 mm	10 Hz
51	3 mm/s	140 W	0 mm	15 Hz
52	3 mm/s	140 W	0 mm	20 Hz
53	3 mm/s	150 W	0 mm	5 Hz
54	3 mm/s	150 W	0 mm	15 Hz
55	3 mm/s	150 W	0 mm	20 Hz
56	3 mm/s	160 W	0 mm	5 Hz
57	3 mm/s	160 W	0 mm	10 Hz
58	3 mm/s	160 W	0 mm	15 Hz
59	3 mm/s	160 W	0 mm	20 Hz
60	3 mm/s	170 W	0 mm	5 Hz
61	3 mm/s	170 W	0 mm	10 Hz
62	3 mm/s	170 W	0 mm	15 Hz
63	3 mm/s	170 W	0 mm	20 Hz
64	3 mm/s	190 W	0 mm	5 Hz
65	3 mm/s	190 W	0 mm	10 Hz
66	3 mm/s	190 W	0 mm	15 Hz
67	3 mm/s	190 W	0 mm	20 Hz
68	3 mm/s	200 W	0 mm	5 Hz
69	3 mm/s	200 W	0 mm	10 Hz
70	3 mm/s	200 W	0 mm	15 Hz
71	3 mm/s	200 W	0 mm	20 Hz
72	3 mm/s	140 W	−1.5 mm	10 Hz
73	3 mm/s	140 W	1.5 mm	10 Hz
74	3 mm/s	150 W	−1.5 mm	10 Hz
75	3 mm/s	150 W	1.5 mm	10 Hz
76	3 mm/s	160 W	−1.5 mm	10 Hz
77	3 mm/s	160 W	1.5 mm	10 Hz
78	3 mm/s	170 W	−1.5 mm	10 Hz
79	3 mm/s	170 W	1.5 mm	10 Hz
80	3 mm/s	190 W	−1.5 mm	10 Hz
81	3 mm/s	190 W	1.5 mm	10 Hz
82	3 mm/s	200 W	−1.5 mm	10 Hz
83	3 mm/s	200 W	1.5 mm	10 Hz
84	3 mm/s	180 W	−1.5 mm	5 Hz
85	3 mm/s	180 W	−1.5 mm	15 Hz
86	3 mm/s	180 W	−1.5 mm	20 Hz
87	3 mm/s	180 W	1.5 mm	5 Hz
88	3 mm/s	180 W	1.5 mm	15 Hz
89	3 mm/s	180 W	1.5 mm	20 Hz
